# The Feasibility of Less-Invasive Bentall Surgery: A Real-World Analysis

**DOI:** 10.3390/life13112204

**Published:** 2023-11-13

**Authors:** Antonia van Kampen, Christian D. Etz, Josephina Haunschild, Martin Misfeld, Piroze Davierwala, Sergey Leontyev, Michael A. Borger

**Affiliations:** 1 Leipzig Heart Center, University Clinic for Cardiac Surgery, Struempellstr. 39, 04289 Leipzig, Germany; 2 Division of Cardiac Surgery, Massachusetts General Hospital, Harvard Medical School, 55 Fruit St., Boston, MA 02114, USA; 3 Department of Cardiac Surgery, Rostock University Hospital, Schillingallee 35, 18057 Rostock, Germany; 4 Department of Cardiothoracic Surgery, Royal Prince Alfred Hospital, 50 Missenden Rd., Camperdown, NSW 2050, Australia; 5 Sydney Medical School, Anderson Stuart Buidling, The University of Sydney, Camperdown, NSW 2050, Australia; 6 Institute of Academic Surgery, Royal Prince Alfred Hospital, 145 Missenden Rd., Camperdown, NSW 2050, Australia; 7 The Baird Institute of Applied Heart and Lung Surgical Research, 100 Carillon Ave., Newtown, NSW 2042, Australia; 8 Division of Cardiac Surgery, Toronto General Hospital, University of Toronto, 585 University Ave., Toronto, ON M5G 2N2, Canada

**Keywords:** aortic aneurysm, aortic root replacement, minimally invasive aortic surgery, Bentall procedure

## Abstract

Objective: Minimally invasive approaches are being used increasingly in cardiac surgery and applied in a wider range of operations, including complex aortic procedures. The aim of this study was to examine the safety and feasibility of a partial upper sternotomy approach for isolated elective aortic root replacement (a modified Bentall procedure). Methods: We performed a retrospective analysis of 768 consecutive patients who had undergone isolated Bentall surgery between January 2000 and January 2021 at our institution, with the exclusion of re-operations, endocarditis, acute aortic dissections, and root replacement with major concomitant procedures such as multi-valve or coronary bypass surgery. A total of 98 patients were operated on via partial sternotomy (PS) and were matched 2:1 to 196 patients operated on via full sternotomy (FS). Results: The procedure time was 12 min longer in the PS group (205 min vs. 192.5 min in the FS group, *p* = 0.002), however, cardiopulmonary bypass and aortic cross-clamp times were comparable between groups. Eight PS-procedures were converted to full sternotomy, predominantly for bleeding complications (*n* = 6). Re-exploration for acute bleeding was necessary in 11% of the PS group and 4.1% of the FS group (*p* = 0.02). Five FS patients and none in the PS group required emergency coronary bypass grafting for postoperative coronary obstruction (*p* = 0.2). PS patients were hospitalized for a significantly shorter period (9.5 days vs. 10.5 days in the FS group, respectively). There were no significant differences regarding in-hospital (*p* = 0.4) and mid-term mortality (*p* = 0.73), as well as for other perioperative complications. Conclusions: Performing Bentall operations via partial upper sternotomy is associated with similar perfusion and cross-clamp times, as well as overall mortality, when compared to a full sternotomy approach. A low threshold for conversion to full sternotomy should be accepted if limited access proves insufficient for the handling of intraoperative complications, particularly bleeding.

## 1. Introduction

The modified Bentall-de Bono procedure includes the replacement of the aortic valve and aortic root, with re-implantation of the coronary ostia into a tubular prosthesis [1]. It is the gold-standard for the treatment of combined pathologies of the aortic valve and aortic root if the cusp anatomy is unfavorable for valve-sparing root replacement [2]. Driven largely by patient preference, minimally invasive strategies for aortic valve and ascending aortic surgery, predominantly performed via partial upper sternotomy, have increased in frequency over the past few decades [3,4]. The use of a limited sternal access for aortic root surgery, however, is not yet an established technique and only very limited evidence on the feasibility and safety of minimally invasive Bentall surgery is currently available [5,6,7,8,9]. The aim of this study was to therefore examine the outcomes of patients who underwent an elective Bentall procedure via partial sternotomy and compare them to those of patients who were operated on via traditional full sternotomy access.

## 2. Materials and Methods

We reviewed our institutional database to identify all adult patients (≥18 years) who had undergone a Bentall procedure between 1 January 2000 and 31 January 2021. Patients with previous cardiac surgery, acute type A aortic dissection or acute endocarditis, and those requiring major concomitant procedures (i.e., coronary bypass surgery, surgery on other heart valves, total aortic arch replacement or MAZE), were excluded. Concomitant procedures that were included consisted of closure of the left atrial appendix, septal myectomy, and replacement of the proximal aortic arch. A total of 768 consecutive patients met the inclusion criteria, and two independent researchers reviewed all electronic health reports.

Supplementary Figure S1 gives an overview of the specific case volume per year at our institution—reasons for the fewer isolated Bentall surgeries in the recent years are (a) increasing numbers of concomitant procedures, and (b) an increasing focus on valve-sparing root repair. Follow-up data were obtained where available from our institution, including surgical reports of re-do procedures during the follow-up period. The study was approved, and individual informed consent waived by the Ethics Committee of the University of Leipzig (177/15).

### 2.1. Indications for Bentall Surgery

In general, indications for aortic root replacement are the relevant disease of the aortic valve (moderate or severe aortic regurgitation or stenosis) and aortic root aneurysms >45 mm. These indications then need to be applied with consideration of individual patient characteristics, and with the evaluation of eligibility for valve-sparing aortic root replacement (David or Yacoub procedures).

### 2.2. Statistical Analysis

Preoperative data and details of surgical technique were compared using a two-group ANOVA (equivalent to *t*-test) or Pearson’s chi-square test with continuity correction, as appropriate, and reported as the mean (plus standard deviation), after confirmation of normal distribution using a Kolmogorov–Smirnov test, or count (percentage). Propensity score 2:1 matching using a “nearest neighbor” algorithm without replacement, and a caliper setting of 1.5 standard deviations, was performed to account for selection bias. Variables included in the propensity score model were age, sex, diabetes, left ventricular ejection fraction, arterial hypertension, pulmonary hypertension, chronic-obstructive pulmonary disease, body mass index, glomerular filtration rate, bicuspid aortic valve, severe aortic regurgitation, peripheral arterial disease, and EuroSCORE II. Standardized mean differences were utilized to evaluate the balancing of covariates after matching. The distribution of propensity scores before and after matching is displayed in Supplementary Figure S2. Due to unequal group sizes, outcome comparisons were performed unpaired, using the Wilcoxon rank-sum test for continuous variables and Fisher’s exact test for categorical variables. Continuous outcomes are reported as medians with interquartile range and categorical data as counts with percentages throughout the manuscript. Data analysis was performed using R version 3.6.1 [10]. Data preparation and statistical analysis were supported by the ‘tidyverse’ [11] package. The ‘MatchIt’ [12] package was used for propensity score modeling and matching, ‘survival’ [13] for survival analysis, and ‘ggplot2’ [14] for plotting.

### 2.3. Surgical Technique

The technical aspects of the minimally invasive Bentall operations have been published by Di Eusanio et al. [15]. Our approach and surgical setup are similar, with some exceptions listed below. The operations were performed either via full sternotomy (FS) or a partial upper sternotomy (PS), using J-, L-, or T-shaped sternal incisions that were extended into the 3rd or 4th intercostal space. Mild hypothermia of 34 °C was the standard temperature management on cardiopulmonary bypass (CPB), although deeper hypothermic regiments (mild to moderate hypothermia) were applied if replacement of the proximal aortic arch was performed (8 (4.1%) of the FS and 6 (6.1%) of the PS group after matching). Arterial cannulas for CPB were usually inserted centrally into the distal ascending aorta or proximal aortic arch; in a few patients, femoral or axillary artery access was chosen to improve access to the surgical field or as safety measure if a large aneurysm was located in close proximity to the sternum. Cannulation of the femoral vein was conducted if visualization of the right atrial appendage was challenging. A left ventricular vent was used in all operations, inserted via the right upper pulmonary vein or, less frequently, pulmonary artery. Blood or crystalloid cardioplegia was delivered into the aortic root or, in case of relevant aortic regurgitation, directly into the coronary ostia. Retrograde application was not used in any of the included patients.

Standard techniques were used to remove the aortic aneurysm and excise the native aortic valve and calcified surrounding tissue. The coronary ostia were preserved as buttons and the proximal coronary arteries were mobilized. Standard valve-bearing conduits used were either commercially or ‘tailor-made’ by the surgeon in the OR by suturing standard valve prostheses into a tubular Dacron graft. Mechanical or biological prostheses were chosen according to current guidelines and patient preferences, along with considering the patient’s individual risk profile. The prostheses used in this cohort are listed in Supplementary Table S1. The coronary buttons were re-implanted using continuous 5-0 polypropylene sutures. Distal anastomosis was usually performed on the cross-clamped aorta. If the proximal aortic arch needed to be replaced, the clamp was removed once the desired level of hypothermia was established, and antegrade selective cerebral perfusion was installed in a uni- or bilateral fashion for an open hemiarch anastomosis. In a standard fashion, patients received one retrosternal (28Fr) and one retrocardial (26Fr) drain, and pleural drains (28Fr) depending on whether the pleura was purposefully (e.g., for mammary artery harvesting) or accidentally opened. This was true for full and partial sternotomies.

## 3. Results

The two matched groups had similar preoperative baseline characteristics, as shown in Table 1. Most patients were male (75.5% in each group, *p* = 1) with a mean age of 59.8 ± 12.2 years in the FS and 60.1 ± 12.2 years in the PS group (*p* = 0.8). Severe aortic stenosis was present in 69.4% of each group (*p* = 1) and severe aortic regurgitation in 41.3% of the FS and 38.8% of the PS group, respectively (*p* = 0.8)—including patients with combined severe valve disease (FS: 8.5%, PS: 8%, *p* = 1). The overall risk profile was similar, with a EuroSCORE II of 4.4 ± 2% in each group (*p* = 0.9). Both elective patient cohorts were therefore of intermediate surgical risk.

Table 2 displays the operative details of the two matched groups. In the PS group, femoral cannulation was used significantly more frequently for venous (FS: 2% vs. 22.4% in the PS group, *p* < 0.001) and arterial access (FS: 1%, PS: 6.1%, *p* = 0.02). In addition, the left ventricular vent was inserted via the pulmonary artery more frequently in the PS group (FS: 1% vs. 19.4% in the PS group, *p* < 0.001). The PS group also received a higher rate of biologic valved conduits (79.6% vs. 62.8% in the FS group, *p* = 0.003). The results of the unmatched groups are displayed in Supplementary Tables S2 and S3.

As shown in Table 3, the overall median procedure time was longer in the PS group (205 [IQR 180–244] vs. 193 [IQR 165–224] min in the FS group, *p* = 0.002), with no significant differences between the FS and PS group in median CPB or aortic cross-clamp times (115 min [IQR 97–135] vs. 111 min [IQR 101–137, *p* = 0.9] and 83 min [IQR 70–98] vs. 84 min [IQR 76–98, *p* = 0.6], respectively). Coronary bypass grafting or venous graft interposition of a coronary vessel (very proximal left main or right coronary artery) was necessary in 4.1% of each group (*p* = 1). There were no significant differences in rates of intraoperative implantation of an intra-aortic balloon pump (FS: 1.5%, PS: 4%, *p* = 0.2) or extracorporeal membrane oxygenation (FS: 1%, PS: 2%, *p* = 0.6). Eight PS operations were converted to full sternotomy: the reasons were bleeding (from proximal anastomosis or coronary buttons) in six cases and emergency CABG in two cases.

There were no significant differences in median total ventilation time (FS: 670 min [IQR 400–975], PS: 525 min [IQR 342–921], *p* = 0.8), length of stay in the intensive care unit (ICU) (FS: 20.5 h [IQR 6.7–26.5], PS: 17.8 h [IQR 5.4–25.3], *p* = 0.2) or length of stay in the intermediate care unit (FS: 35.7 h [IQR 19.5–70.8], PS: 37.2 h [IQR 21.1–71], *p* = 0.5). Surgical re-exploration was necessary for bleeding in 4.1% of the FS and 11.2% of the PS group (*p* = 0.02) and for hemodynamically relevant pericardial effusion in 8.7% of the FS and 5.1% of the PS group (*p* = 0.4). When comparing earlier cases (before vs. since 2010), a clear learning curve effect was observed: before 2010, re-exploration for bleeding was performed in 14.5% of the PS and 3.4% of the FS group, and since 2010 the rates have been 5.6% and 2.6%, respectively. Of note, only one patient who was converted from PS to FS required postoperative re-exploration for bleeding and none of the converted patients died during hospitalization.

Five patients (1.7%) of the FS group required emergency re-operation and CABG for a postoperative coronary obstruction versus 0% in the PS group, *p* = 0.2. Sternal re-fixation due to instability was necessary in 2% of the FS and 1% of the PS group (*p* = 0.5). No statistically significant differences were detected for stroke, pacemaker implantations, and respiratory failure requiring re-intubation. There was a trend towards more postoperative dialysis in the PS group (10.2% vs. 4.1% in the FS group, *p* = 0.07). Patients operated on via PS were hospitalized for significantly shorter periods than those operated on via FS (9.5 days [IQR 8–12] vs. 10.5 days [IQR 9–15], *p* = 0.02).

**Figure 1:**
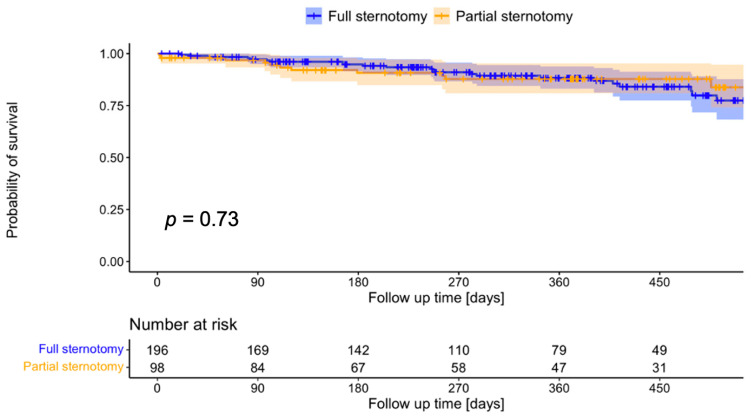
Kaplan–Meier analysis of survival (log-rank test).

There were four cases of in-hospital death (4%) in the PS group and four (2%) in the FS group (*p* = 0.4). Of note, all eight in-hospital-deaths occurred before 2009. Reasons were low cardiac output in four, septic multi organ failure in three, and brain stem hemorrhage in one patient. Kaplan–Meier analysis (Figure 1) did not detect a difference in mid-term survival via the log-rank test (*p* = 0.73).

We further analyzed the PS patients in detail and the results are displayed in Table 4. The partial upper sternotomy was conducted as a T-shaped incision in 22 patients (22.4%), and a J- or L-shaped incision in 76 patients (77.6%). Although the numbers were low and the significance level was never reached, trends towards more intra- and postoperative complications in the J/L group than the T group were observed. Most importantly, 13.2% of the J/L group and only 4.5% of the T group required re-exploration for bleeding. Furthermore, all the ECMO, IABP, pacemaker, stroke, and in-hospital death incidences occurred in the J/L group.

## 4. Discussion

We compared patients undergoing aortic root and valve replacement (i.e., a Bentall procedure) via full vs. partial sternotomy, and did not find significantly increased CPB or aortic cross-clamp times with the minimally invasive approach. However, total operating times were 13 min longer in the PS group, probably because of the technical challenges of operating through a small incision. Biological valve prostheses were used more frequently in the PS group, yet the reasons for this are not entirely clear.

A meta-analysis of all the studies published on minimally invasive aortic root replacement by Harky et al. concluded that partial sternotomy was a safe approach and was even associated with shortened CPB times. However, the informative value of their analysis was limited due to the marked heterogeneity of the included articles—some had included valve-sparing root replacement patients with unequal proportions between the full and partial sternotomy groups and without any matched analyses [16]. Furthermore, minimally invasive procedures are, in general, more often performed by more experienced surgeons, frequently in “high-volume” centers, and this needs to be considered when comparing procedural times, e.g., CPB time.

Three studies that included patients undergoing minimally invasive Bentall operations, which only consisted of very small patient cohorts (31 to 35 each), reported on cross-clamp times of 113–157 min and CPB times of 162–193 min, which is considerably longer than those observed in our PS group (83.5 and 110.5 min on average, respectively) [6,7,9]. Mikus et al. reported a mean cross-clamp time of 74 and mean CPB time of 84 min in 53 Bentall patients operated on via partial sternotomy [8]. The largest minimally invasive Bentall cohort yet, containing 72 patients, was published by Levack et al. as part of a comprehensive single-center study on 568 patients undergoing a wide range of proximal aortic operations via partial sternotomy. Yet, their outcome analysis did not differentiate between surgical procedures, thus the specific outcomes of their minimally invasive Bentall patients remain unclear and cannot be compared to the presented data [5].

More patients in our PS group (11.2% vs. 4.1%, *p* = 0.02) required surgical re-exploration for bleeding in the acute postoperative period. When comparing patients operated on before vs. after 2010, however, we observed a marked decrease in this complication in the PS group over time (14.5% before vs. 5.6% after 2010). Such a finding is suggestive of a learning curve, which is known to exist for most complex surgical procedures. In the abovementioned small cohort studies on minimally invasive Bentall surgery, rates of re-exploration for bleeding ranged from 3.1% to 6.1% (5, 6, 8), which is comparable to the rate detected in our cohort between 2010 and 2021. An observational study by Fröjd and Jepssen of 5293 patients undergoing cardiac surgery found re-exploration for bleeding to be independently associated with the need for postoperative dialysis [17], and hemorrhage is a known risk factor for acute renal failure after cardiac surgery [18,19]. These are possible explanations for the trend towards an increased rate in postoperative dialysis in our PS group.

Eight PS operations were converted to full sternotomies in our cohort, six for bleeding complications and two for emergency CABG due to coronary malperfusion. Only one of the converted patients required re-exploration for bleeding, and none of them required dialysis or died in hospital after the operation. We therefore recommend a relatively low threshold for conversion to full sternotomy, particularly if the patient’s anatomy does not appear suitable for minimally invasive surgery or complications occur that can best be managed with unrestricted access to the entire operative field. None of the previously published studies on minimally invasive Bentall surgery reported sternal conversion rates, making a comparison of our results with other studies difficult. Levack et al. reported an overall conversion rate of 1.9% in their minimally invasive aortic surgery cohort, but did not provide data for the Bentall subgroup specifically [5].

Although patient preference and competition between cardiac surgical programs are likely to drive further increases in minimal-access techniques, patient safety must always be the priority. Any compromise of a safe and durable treatment result in favor of a cosmetically (or psychologically) more desirable ‘shorter incision’ should be avoided. Conversion from partial to full sternotomy—if necessary—is uncomplicated, easy to perform, and can contribute significantly to patient safety and improved surgical outcomes.

To minimize the risk of conversion, however, and improve intra- and postoperative outcomes, patient selection and thorough preprocedural planning is of the outmost importance. Regarding anatomic considerations, we recommend particular attention to an elongated ascending aorta, as well as a very caudally located or horizontally oriented aortic root, as these may impair exposure of the surgical site and potentially complicate exposure via partial sternotomy. Similarly, caution is required if the mediastinum is markedly shifted to the left or right side, although a T-shaped sternal incision might still allow for a minimally invasive approach. Before matching, in this cohort, there was significantly more severe aortic regurgitation in the FS group, potentially hinting at surgeons selecting more comfortable access for direct ostial cardioplegia delivery. In any case, flexibility in respect to the intraoperative setup is a cornerstone of a successful minimally invasive cardiac surgery program. For example, the right atrial venous cannula can be tunneled out via an additional skin incision to pull away the right atrial appendage and better expose the aortic root and right coronary artery. Femoral venous cannulation can also be utilized to improve venous drainage and minimize the amount of material in the operative field. If soft plaques and calcifications are ruled out via CT examination of the aorta, iliac, and femoral vessels, arterial access can also be established via the femoral artery without increasing the risk of stroke [20]. If surgery on the aortic arch is required, cannulation of the axillary artery is a reasonable option, as it supports the safe and rapid establishment of unilateral antegrade selective cerebral perfusion and facilitates early distal perfusion if combined with an “arch first” strategy without additional tubing in the field (i.e., an additional perfusion branch).

Patients who may benefit from a minimally invasive strategy are those that suffer from chronic lung disease and are at risk for pulmonary complications, and possibly those with osteoporosis/osteopenia or other risk factors for impaired sternal healing. We were not able to show an advantage of a PS regarding pulmonary or sternal complications, possibly due to the relatively small group sizes and low event rates.

A right-anterolateral mini-thoracotomy approach is increasingly being used in a growing number of institutions for aortic valve replacement surgery [21]. With increasing experience, more complex operations might become feasible through this approach. The first promising results of sternal-sparing Bentall procedures have already been published by Johnson et al. [22]. Preserving sternal integrity could further accelerate postoperative mobilization and regeneration, making thoracotomy incisions an interesting area for experienced surgeons with specific training.

The current study has some limitations, most importantly in its retrospective design. Propensity score matching only accounts for recorded variables and the likelihood of selection bias remains. Furthermore, we examined patients that were operated on over the course of two decades. Advances in surgical technique and perioperative care might have influenced outcomes, as well as changing staff at our institution. As depicted in Figure 1, the Bentall procedure has been less frequently performed as an isolated procedure at our institution over time, reducing the applicability of a minimally invasive approach.

Despite these limitations, we herein present the largest minimally invasive Bentall cohort to date, with very satisfying results. Although the minimally invasive Bentall operation remains a technically challenging procedure and should probably be reserved for high-volume aortic centers and surgeons, we conclude that it is a safe and feasible option for select patients with combined aortic root and aortic valve disease.

## Figures and Tables

**Table 1:** Baseline characteristics of the unmatched and 2:1 matched groups.

	Unmatched					Matched				
	Total *n* = 768	FS *n* = 670	PS *n* = 98	*p*-Value	SMD	Total *n* = 294	FS *n* = 196	PS *n* = 98	*p*-Value	SMD
Age	59.4 ± 12.2	59.3 ± 12.2	60.1 ± 12.2	0.5	0.066	59.8 ± 12.2	59.7 ± 12.2	60.1 ± 12.2	0.8	0.038
Male gender	596 (77.6)	522 (77.9)	74 (75.5)	0.7	0.057	222 (75.5)	148 (75.5)	74 (75.5)	1	0
BMI	27.8 ± 4.6	27.9 ± 4.6	27.2 ± 4.7	0.1	0.161	27.1 ± 4.6	27.1 ± 4.6	27.2 ± 4.7	0.9	0.01
Art. HTN	584 (76)	515 (76.9)	69 (70.4)	0.2	0.147	206 (70.1)	137 (69.9)	69 (70.4)	1	0.011
Pulm. HTN	85 (11.1)	71 (10.6)	14 (14.3)	0.4	0.112	39 (13.3)	25 (12.8)	14 (14.3)	0.9	0.045
Diabetes	83 (10.8)	75 (11.2)	8 (8.2)	0.5	0.103	21 (7.1)	13 (6.6)	8 (8.2)	0.8	0.059
COPD	27 (3.5)	25 (3.7)	2 (2)	0.6	0.101	6 (2)	4 (2)	2 (2)	1	0
PAD	411 (53.5)	367 (54.8)	44 (44.9)	0.09	0.199	132 (44.9)	88 (44.9)	44 (44.9)	1	0
eGFR	97.4 (32.6)	97.8 (32.7)	94 (31.5)	0.3	0.118	93.9 (30.6)	94.7 (30.9)	92.5 (30)	0.6	0.072
LVEF	57.4 ± 12	57.1 ± 12.1	59.1 ± 11	0.1	0.038	59.4 ± 11.9	59.6 ± 12.5	59.2 ± 10.6	0.8	0.035
BAV	358 (46.6)	314 (46.9)	44 (44.9)	0.8	0.039	138 (46.9)	94 (48)	44 (44.9)	0.7	0.061
severe AS	358 (46.6)	290 (43.3)	68 (69.4)	**<0.001**	0.546	204 (69.4)	136 (69.4)	68 (69.4)	1	0
severe AR	456 (59.4)	418 (62.4)	38 (38.8)	**<0.001**	0.486	119 (40.5)	81 (41.3)	38 (38.8)	0.8	0.052
Euro-SCORE II	4.7 ± 2.1	4.7 ± 2.1	4.4 ± 2.1	0.3	0.121	4.4 ± 2	4.4 ± 2	4.4 ± 2	0.9	0.022

Continuous variables presented as means with standard deviations, and compared using ANOVA for unmatched and pWilcoxon sign rank test for matched comparisons, categorical variables presented as numbers with percentages, compared using Pearson’s chi-squared test for unmatched and McNemar’s test for matched comparisons. Bold *p*-values indicate statistical significance with an alpha-level of 0.05. AR = aortic regurgitation; AS = aortic stenosis; BAV = bicuspid aortic valve; BMI = body mass index; COPD = chronic obstructive pulmonary disease; eGFR = estimated glomerular filtration rate (Cockgroft–Gault formula); FS = full sternotomy; HTN = hypertension LVEF = left-ventricular ejection fraction; PAD = peripheral artery disease; PS = partial sternotomy; SMD = standardized mean difference.

**Table 2:** Details of operative techniques.

Matched Cohorts				
	Total *n* = 294	FS *n* = 196	PS *n* = 98	*p*-Value
Arterial cannulation				
Central	280 (95.2)	188 (95.9)	92 (93.9)	0.6
Femoral	8 (2.7)	2 (1)	6 (6.1)	**0.02**
Axillary	6 (2)	6 (3.1)	0 (0)	0.2
Venous cannulation				
Central	268 (91.2)	192 (98)	76 (77.6)	**<0.001**
Femoral	26 (8.8)	4 (2)	22 (22.4)	**<0.001**
LV vent				
via pulmonary vein	274 (93.2)	195 (99.5)	79 (80.6)	**<0.001**
via pulmonary artery	20 (6.8)	1 (0.5)	19 (19.4)	**<0.001**
Concomitant procedures				
LAA occlusion	2 (0.7)	2 (1)	0 (0)	0.6
morrow resection	15 (5.1)	8 (4.1)	7 (7.1)	0.3
hemiarch replacement	14 (4.8)	8 (4.1)	6 (6.1)	0.7
Biological valve prosthesis	201 (68.4)	123 (62.8)	78 (79.6)	**0.003**

Categorical variables presented as numbers with percentages, compared using McNemar’s test. Bold *p*-values indicate statistical significance with an alpha-level of 0.05. LAA = left atrial appendix; LV = left ventricle; FS = full sternotomy; PS = partial sternotomy.

**Table 3:** Intraoperative and postoperative outcomes.

	Matched Cohorts				
	Total	FS	PS	OR (95% CI)	*p*-Value
	*n* = 294	*n* = 196	*n* = 98		
Procedure time [min]	197 (171.3–230)	192.5 (165–224)	205 (180–243.8)	-	**0.002**
Bypass time [min]	113.5 (98.3–136)	114.5 (96.8–135.3)	110.5 (101–137)	-	0.9
Aortic cross-clamp time [min]	86 (74.3–99.8)	87 (73.8–102)	83.5 (76–97.75)	-	0.6
Median prosthesis size	27 (25;27)	25 (25;27)	27 (25;27)	-	0.4
Conversion	-	-	8 (8.2)	-	-
Emergency CABG	12 (4.1)	8 (4.1)	4 (4.1)	1 (0.2–3.8)	1
IABP	8 (2.7)	4 (2)	4 (4)	2 (0.4–11.2)	0.4
ECMO	4 (1.4)	2 (1)	2 (2)	1.3 (0.1–11.9)	1
Ventilation time [minutes]	567 (372–942)	670 (400–975)	525 (342–921)	-	0.2
ICU length of stay [hours]	19.9 (6.3–26.3)	20.5 (6.7–26.5)	17.8 (5.4–25.3)	-	0.2
IMCU length of stay [hours]	36.3 (20–70.9)	35.7 (19.5–70.8)	37.2 (21.1–71)	-	0.7
Revision f. bleeding	19 (6.5)	8 (4.1)	11 (11.2)	3 (1.04–8.8)	**0.02**
Revision f. pericardial effusion	22 (7.5)	17 (8.7)	5 (5.1)	0.6 (0.2–1.7)	0.4
Revision f. coronary compl.	5 (1.7)	5 (2.6)	0 (0)	0 (0–2.2)	0.2
Revision f. sternal instability	5 (1.7)	4 (2)	1 (1)	0.5 (0.01–5.1)	0.5
GI complications	14 (4.7)	11 (5.6)	3 (3.1)	0.5 (0.09–2.1)	0.4
Stroke	5 (1.7)	2 (1)	3 (3.1)	3.05 (0.3–37.1)	0.3
Pacemaker	10 (3.4)	8 (4.1)	2 (2)	0.5 (0.05–2.5)	0.5
Respiratory failure	36 (6.1)	26 (13.3)	10 (10.2)	0.7 (0.3–1.7)	0.6
Dialysis	18 (3.1)	8 (4.1)	10 (10.2)	2.7 (0.9–8)	0.07
In-hospital death	8 (2.7)	4 (2)	4 (4.1)	2 (0.4–11.2)	0.4
Hospitalization [days]	10 (8–14)	10.5 (8.8–15)	9.5 (8–12)	-	**0.02**

Continuous variables presented as medians with interquartile ranges, and compared using Wilcoxon sign rank test, categorical variables presented as numbers with percentages, compared using McNemar’s test. Bold *p*-values indicate statistical significance with an alpha-level of 0.05. CABG = coronary artery bypass grafting; ECMO = extracorporeal membrane oxygenation; FS = full sternotomy; GI = gastrointestinal; IABP = intra-aortic balloon pump; ICU = intensive care unit; IMCU = intermediate care unit; OR = odds ratio; PS = partial sternotomy.

**Table 4:** Detailed analysis of the minimally invasive procedures.

	Minimally Invasive Operations Only				
	Total	T-Incision	J/L-Incision	OR (95% CI)	*p*-Value
	*n* = 98	*n* = 22	*n* = 76		
Procedure time [minutes]	205 (180–244)	205 (190–229)	206 (179–245)	-	0.9
Bypass time [minutes]	111 (101–137)	117 (107–145)	110 (96–133)	-	0.3
Aortic cross-clamp time [minutes]	84 (76–98)	81 (76–94)	85 (76–99)	-	0.6
Conversion to full sternotomy	8 (8)	2 (9.1)	6 (7.9)	1.2 (0.1–7.4)	1
Revision for bleeding	11 (11.2)	1 (4.5)	10 (13.2)	0.3 (0.01–2.5)	0.5
Revision for pericardial effusion	5 (5)	1 (4.5)	4 (5.3)	0.9 (0.02–9.3)	1
Revision for sternal instability	1 ()	0 (0)	1 (1.3)	0 (0–19)	1
ECMO	2 (2)	0 (0)	2 (2.6)	0 (0–19)	1
IABP	4 (4)	0 (0)	4 (5.3)	0 (0–5.3)	0.6
Pacemaker	2 (2)	0 (0)	2 (2.6)	0 (9–19)	1
Stroke	3 (3)	0 (0)	3 (3.9)	0 (0–8.5)	1
In-hospital death	4 (4)	0 (0)	4 (5.3)	0 (0–5.3)	0.6

Continuous variables presented as medians with interquartile ranges, and compared using Wilcoxon sign rank test, categorical variables presented as numbers with percentages, compared using McNemar’s test. ECMO = extracorporeal membrane oxygenation; IABP = intra-aortic balloon pump; OR = odds ratio.

## Data Availability

The data included in this manuscript cannot be shared publicly, due to the need to protect the privacy of the included subjects. Data may be shared upon reasonable request to the corresponding author.

## Supplementary Materials

The following supporting information can be downloaded at: https://www.mdpi.com/article/10.3390/life13112204/s1, Table S1: Prostheses used in the matched cohorts; Table S2: Intraoperative details of the unmatched cohorts; Table S3: Postoperative outcomes of the unmatched cohorts. Figure S1: Case volumes; Figure S2: PS distribution plot.
