# Supplementary material for: The Feasibility of Less-Invasive Bentall Surgery: A Real-World Analysis
Source: Life (Basel). 2023 Nov 13;13(11):2204. doi: 10.3390/life13112204 (PMC10671842; doi:10.3390/life13112204)
Supplement: Supplementary file 1 [file life-13-02204-s001.zip › Supplemental Table S1 (Prosthesis types).pdf]

Supplemental Table S1: Prostheses used in the matched cohorts

Matched cohorts					
		**Total n=294**	**FS n=196**	**PS n=98**	**p-value**
**Mechanical prostheses**	***n (%)***	**93 (31.6)**	**74 (37.8)**	**19 (19.4)**	**0.003**
Mechanical valved conduits					
ATS Aortic Valved Graft	*n (%)*	76 (25.9)	58 (29.6)	18 (18.4)	
SJM Aortic Valved Graft	*n (%)*	15 (5.1)	14 (4.8)	1 (1)	
Used as "tailor-made" mechanical conduit					
ATS Open Pivot	*n (%)*	2 (0.7)	2 (1)	0 (0)	
**Biological prostheses**	***n (%)***	**201 (68.4)**	**123**	**78 (79.6)**	**0.003**
Biological valved conduits					
Medtronic Freestyle	*n (%)*	73 (24.8)	41 (20.9)	32 (32.7)	
SJM Toronto Root	*n (%)*	50 (17)	24 (12.2)	26 (26.5)	
Vascutek BioValsalva	*n (%)*	7 (2.4)	7 (3.6)	0 (0)	
Used as "tailor-made" biological conduit					
CE Perimount Magna-Ease	*n (%)*	8 (2.7)	6 (3.1)	2 (2)	
CE Perimount	*n (%)*	61 (20.7)	43 (21.9)	18 (18.4)	
Medtronic Avalus	*n (%)*	1 (0.3)	0 (0)	1 (1)	
Homografts	*n (%)*	1 (0.5)	0 (0)	1 (1)	

CE=Carpentier-Edwards; FS=full sternotomy; PS=partial sternotomy; SJM=St.Jude Medical
