# Supplementary material for: The Feasibility of Less-Invasive Bentall Surgery: A Real-World Analysis
Source: Life (Basel). 2023 Nov 13;13(11):2204. doi: 10.3390/life13112204 (PMC10671842; doi:10.3390/life13112204)
Supplement: Supplementary file 1 [file life-13-02204-s001.zip › Supplemental Table S2 (intraop. unmatched cohorts).pdf]

Supplemental Table S2: Intraoperative details of the unmatched cohorts

		Unmatched cohorts			
		**Total n=768**	**FS n=670**	**PS n=98**	**p-value**
Arterial cannulation					
Central	*n (%)*	738 (96)	646 (96.4)	92 (93.9)	0.3
Femoral	*n (%)*	15 (2)	9 (1.3)	6 (6.1)	**0.007**
Axillary	*n (%)*	15 (2)	15 (2.2)	0 (0)	0.2
Venous cannulation					
Central	*n (%)*	738 (96	662 (98.8)	76 (77.6)	**<0.001**
Femoral	*n (%)*	30 (3.9)	8 (1.2)	22 (22.4)	**<0.001**
LV-vent					
via pulmonary vein	*n (%)*	747 (97.3)	668 (99.7)	79 (80.6)	**<0.001**
via pulmonary artery	*n (%)*	21 (2.7)	2 (0.3)	19 (19.4)	**<0.001**
Concomitant procedures					
LAA occlusion	*n (%)*	18 (2.3)	18 (2.7)	0 (0)	0.6
morrow resection	*n (%)*	32 (4.2)	25 (3.7)	7 (7.1)	0.07
proximal-arch replacement	*n (%)*	21 (2.7)	15 (2.2)	6 (6.1)	0.2
Biological valve prosthesis	*n (%)*	507 (66)	429 (64)	78 (79.6)	**0.001**

LAA=left atrial appendix; LV=left ventricle; FS=full sternotomy; PS=partial sternotomy
Data compared using Pearson’s chi-squared test. Bold p-values are <0.05, indicating statistical significance.
