# Supplementary material for: The Feasibility of Less-Invasive Bentall Surgery: A Real-World Analysis
Source: Life (Basel). 2023 Nov 13;13(11):2204. doi: 10.3390/life13112204 (PMC10671842; doi:10.3390/life13112204)
Supplement: Supplementary file 1 [file life-13-02204-s001.zip › Supplemental Table S3 (postop. unmatched cohorts).pdf]

Supplemental Table S3: Postoperative outcomes of the unmatched cohorts

		Unmatched cohorts				
		**Total n=768**	**FS n=670**	**PS n=98**	**OR (95%-CI)**	**p-value**
Procedure time [min]	*median (IQR)*	189 (161-222)	185 (160-220)	205 (180-243.8)	-	**0.008**
Bypass time [min]	*median (IQR)*	110 (93-133)	110 (92-132)	110.5 (100.5-137)	-	0.1
Aortic cross-clamp time [min]	*median (IQR)*	85 (71-99)	85 (70.3-98.8)	83.5 (76-97.75)	-	0.6
Conversion	*n (%)*	-	-	8 (8.2)	-	-
Emergency CABG	*n (%)*	19 (2.5)	15 (2.2)	4 (4.1)	1.9 (0.4-6)	0.3
IABP	*n (%)*	11 (1.4)	7 (1)	4 (4)	4 (0.8-16.2)	**0.04**
ECMO	*n (%)*	6 (0.8)	4 (0.6)	2 (2)	3.5 (0.3-24.5)	0.2
Ventilation time [minutes]	*median (IQR)*	570 (360-969)	576.3(360-970.2)	525 (342-921)	-	0.5
ICU length of stay [hours]	*median (IQR)*	19.5 (6.6-25.2)	19.7(6.8-25.2)	17.8 (5.4-25.3)	-	0.5
IMCU length of stay [hours]	*median (IQR)*	36.9 (21-72)	36.8 (21-72)	37.2 (21.1-71)	-	0.9
Revision for bleeding	*n (%)*	42 (5.5)	31 (4.6)	11 (11.2)	2.6 (1.1-5.6)	**0.01**
Revision for pericardial effusion	*n (%)*	63 (8.2)	58 (8.7)	5 (5.1)	0.6 (0.2-1.5)	0.3
Revision for coronary complications	*n (%)*	8 (1)	8 (1.2)	0 (0)	0 (0-4)	0.6
Revision for sternal instability	*n (%)*	8 (1)	7 (1)	1 (1)	1 (0.02-7.7)	1
GI complications	*n (%)*	32 (4.2)	29 (4.3)	3 (3.1)	0.7 (0.1-2.3)	0.8
Stroke	*n (%)*	13 (1.7)	10 (1.5)	3 (3.1)	2.1 (0.4-8.3)	0.2
Pacemaker	*n (%)*	36 (4.7)	34 (5)	2 (2)	0.4 (0.04-1.6)	0.3
Respiratory failure	*n (%)*	72 (9.4)	62 (9.3)	10 (10.2)	1.1 (0.5-2.3)	0.7
Dialysis	*n (%)*	34 (4.4)	24 (3.6)	10 (10.2)	3.1 (1.3-6.9)	**0.007**
In-house death	*n (%)*	15 (2)	9 (1.3)	4 (4.1)	3.1 (0.7-11.5)	0.07
Hospitalisation [days]	*median (IQR)*	11 (8-14)	11 (8-14)	9.5 (8-12)	-	**0.02**

CABG=coronary artery bypass grafting; ECMO=extracorporeal membrane oxygenation; FS=full sternotomy; GI=gastrointestinal; IABP=intraaortic balloon pump; ICU=intensive care unit;IMCU=intermediate care unit;OR=odds ratio; PS=partial sternotomy
Data compared using Wilcoxon rank sum test or Fisher's exact test. Bold p-values are <0.05, indicating statistical significance
