# Supplementary figures and images for: The Feasibility of Less-Invasive Bentall Surgery: A Real-World Analysis
Source: Life (Basel). 2023 Nov 13;13(11):2204. doi: 10.3390/life13112204 (PMC10671842; doi:10.3390/life13112204)

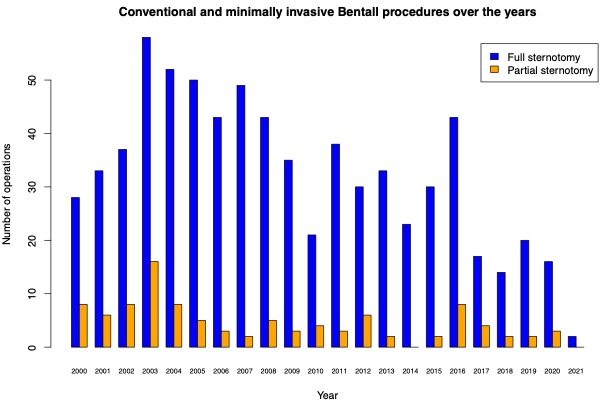

Supplement: Supplementary file 1 [file life-13-02204-s001.zip › Suppl. Figure S1. case volumes.jpg]

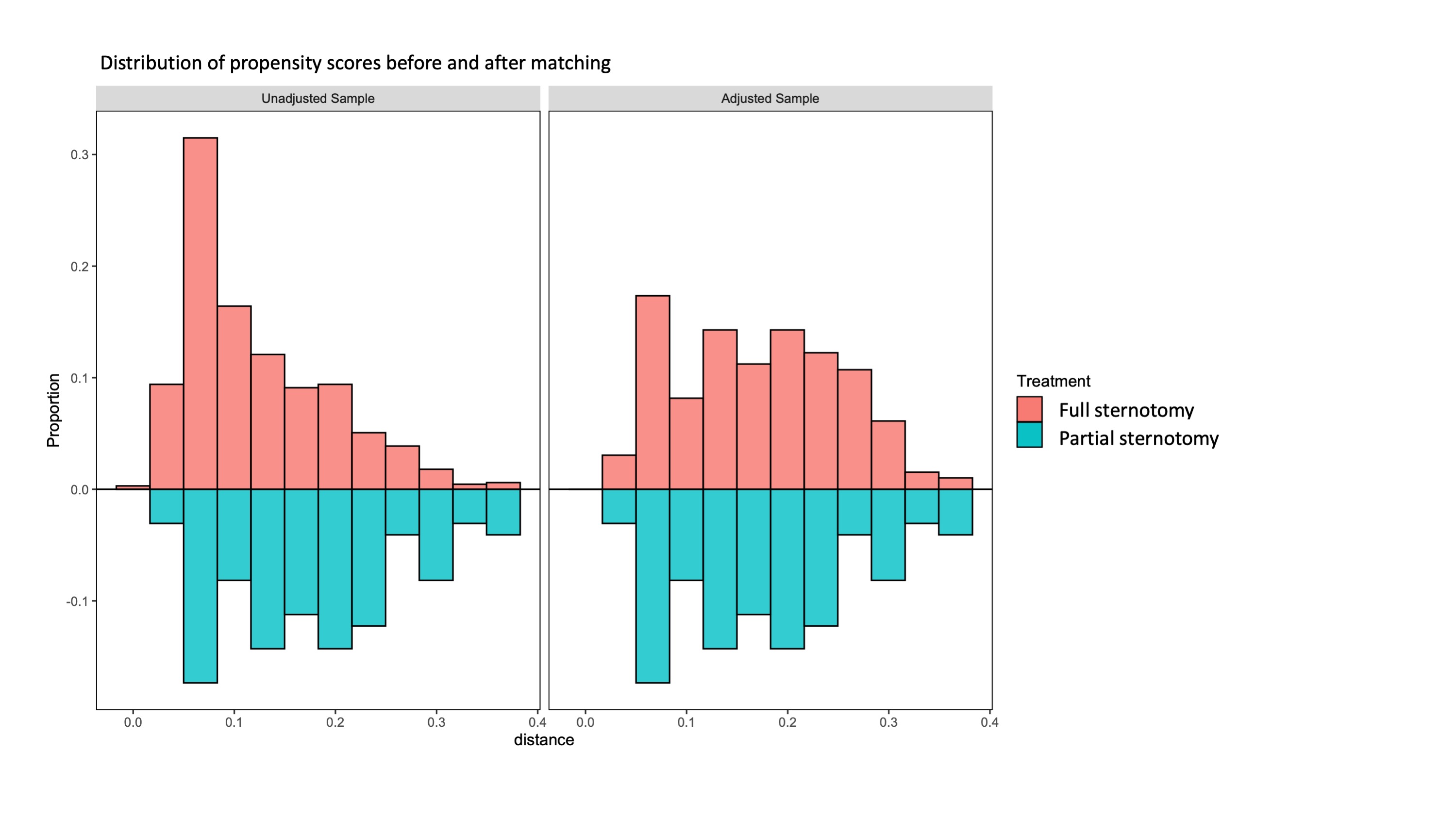

Supplement: Supplementary file 1 [file life-13-02204-s001.zip › Suppl. Figure S2. PS distribution plot.jpg]
